# Abnormally Long O–O Bond in *trans*-HOON: An Exemplary Charge-Shift Bond

**DOI:** 10.1021/acs.jpca.5c02743

**Published:** 2025-06-25

**Authors:** Huaiyu Zhang, Jia Wei, Rui Ma, Jinshuai Song, Wei Wu, Yirong Mo

**Affiliations:** † Institute of Computational Quantum Chemistry, and Hebei Key Laboratory of Inorganic Nano-materials, College of Chemistry and Materials Science, 66447 Hebei Normal University, Shijiazhuang 050024, China; ‡ Green Catalysis Center, and College of Chemistry, 12636 Zhengzhou University, Zhengzhou 450001, China; § State Key Laboratory of Physical Chemistry of Solid Surfaces, Fujian Provincial Key Laboratory of Theoretical and Computational Chemistry, and College of Chemistry and Chemical Engineering, 12466 Xiamen University, Xiamen 361005, China; ∥ Department of Nanoscience, Joint School of Nanoscience and Nanoengineering, 14616 University of North Carolina at Greensboro, Greensboro, North Carolina 27401, United States

## Abstract

Nitrous acid (HONO) plays a significant role in atmospheric and combustion chemistry. While extensive attention has been devoted to the study of HONO, its isomer (HOON) has remained relatively unexplored until recent experimental and theoretical analyses revealed its unusually long and weak O–O bond. In contrast, its sulfur-substituted analogue, HOSN, exhibits a normal O–S bond. Here, we explored the intriguing bonding nature of *trans*-HOXN (X = O, S) from the perspective of the ab initio valence bond (VB) theory in order to elucidate the different behaviors of the O–O and O–S bonds therein. Our results demonstrated that the bonding in *trans*-HOON can be described as a three-center four-electron charge-shift bond, where the ON moiety most closely resembles nitric oxide, with some nitrene characters. Since the π bond in ON is a dative bond resulting from one lone pair on the oxygen atom, the accumulated negative charge on N enhances the hyperconjugation from the nitrogen lone pair of σ symmetry to the σ*_O–O_ orbital. Ultimately, it is the enhanced hyperconjugative interaction that plays a dominating role in the elongation and weakening of the O–O bond. In contrast, *trans*-HOSN is characterized as a two-center two-electron charge-shift bond. Compared with H_2_O_2_ which takes a skew geometry, both *trans*-HOXN (X = O, S) prefer a planar geometry. While geometric relaxation provides the primary stabilizing force for *trans*-HOON, the planarity of *trans*-HOSN arises dominantly from the conjugation and hyperconjugation effects.

## Graphical abstract

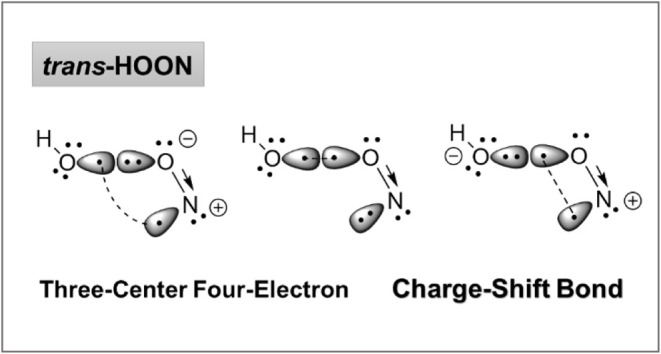

## Introduction

Nitrous acid (HONO) plays a pivotal role in both atmospheric and combustion chemistry and has attracted considerable attention.^1–5^ However, research on the isomer HOON remains limited. Experimentally, the existence of HOON was unknown until Crabtree *et al.* reported the Fourier transform microwave spectrum of *trans*-HOON in 2013.^6^ A striking feature of *trans*-HOON is its unusually long O–O bond length of 1.9149 ± 0.0005 Å,^6^ which is significantly elongated (by 0.45 Å) compared with the O–O single bond in peroxides R_2_O_2_ whose O–O bond lengths are around 1.45–1.50 Å. Most recently, Li *et al.* directly observed HOON in the photochemistry of HONO, employing matrix-isolation IR and UV–vis spectroscopy.^7^ Their findings underscore the significance of HOON as a key intermediate in the photolytic dissociation–association cycle of HONO at low temperatures.

Theoretically, although HOON has been mentioned in a few studies,^8–11^ the reported O–O bond length varied from 1.47 to 2.03 Å until Talipov *et al.* confirmed the long and weak O–O bond in *trans*-HOON by high-level single- and multireference *ab initio* calculations.^12^ Based on the topological analysis of the electron density, they pointed out that HOON can be best represented as a combination of three resonance structures, with the major contribution from a radical-pair structure, followed by a significant contribution from a nitrene structure and a minor admixture of ion-pair character. In contrast to *trans*-HOON, its sulfur-substituted analogue, *trans*-HOSN, exhibits a normal O–S bond length and a dissociation energy comparable to HOSH.^13–15^ Subsequently, Takeshita and Dunning performed generalized valence bond (GVB) calculations to explore the bonding nature of *trans*-HOXN (X = O, S).^16^ Their calculations revealed that the long O–O bond in *trans*-HOON results from a weak through-pair interaction, while the O–S bond in *trans*-HOSN arises from the formation of a stable recoupled pair bond dyad.

To shed new light on the unusual structure of HOON, alternative computational approaches apart from popular molecular orbital (MO) or DFT methods are expected. In terms of bonding analyses, a classical valence bond (VB) theory^17,18^ stands out as it can provide unparalleled conceptual insights into the bonding nature of compounds and the dominant resonance structures, particularly when combined with rigorous *ab initio* calculations. Here, we conducted a new study on the electronic structures of *trans*-HOXN (X = O, S) based on the classical ab initio VB method. Apart from the quantification of the hyperconjugative interaction in HOON, our study showed that such a hyperconjugative interaction can be better described in terms of the charge-shift bond.

## Theoretical Methods and Computational Details

In the VB theory,^17,18^ a many-electron system is described with a set of resonance structures, and each resonance structure can be defined with a Heitler–London–Slater–Pauling (HLSP) function. Accordingly, the molecular wave function Ψ is a linear combination of HLSP functions as

1 $$\Psi =\underset{K}{\sum }C_{K}\Phi_{K}$$

where $\Phi_{K}$ corresponds to a “classical” VB structure *K* and *C_K_* is its structural coefficient. In our VB calculations, all σ orbitals are strictly localized either between two bonding atoms or on single atoms as lone pairs to ensure a clear correspondence between the mathematical expressions of the VB structures and their physical meanings. Unless otherwise specified, however, π electrons are delocalized. The contributions of the VB structures can be evaluated based on their structural weights using the Coulson–Chirgwin formula (eq 2),^19^ which is the equivalent of a Mulliken population analysis in the MO theory as

2 $$W_{K}=C_{K}^{2}+\underset{L\neq K}{\sum }C_{K}C_{L}S_{KL}$$

where *S_KL_* is the overlap integral between two VB structures *K* and *L*.

In the VB self-consistent-field (VBSCF)^20^ procedure, both the VB orbitals and structural coefficients are optimized simultaneously to minimize the total energy. The VBSCF method includes static electron correlations to some extent but lacks dynamic correlations. This is because all structures are constructed with the same set of orbitals. A significant improvement is the use of the breathing orbital VB (BOVB) method,^21,22^ in which each structure is constructed with its own set of optimal orbitals. In this work, we utilized the BOVB method.

Throughout this work, geometry optimizations were carried out at the CCSD­(T) theoretical level as implemented in the Gaussian 16 program.^23^ Harmonic vibrational calculations were performed at the same level to assess the nature of the stationary points on the potential energy surfaces. The VB calculations were carried out with the XMVB code,^24^ which is an ab initio VB program. The Multiwfn package^25^ was used to perform the adaptive natural density partitioning (AdNDP) analysis^26,27^ by postprocessing the wave function files generated from Gaussian 16 calculations. The magnitude of hyperconjugation was measured from a second-order perturbation analysis implemented in the NBO program.^28^ The aug-cc-pVTZ basis sets^29–31^ were used, excluding the f functions of the sulfur atom to facilitate convergence in VB calculations.

## Results and Discussion

### Geometries and AdNDP Analysis of *trans*-HOXN (X = O, S)

The optimized geometries of *trans*-HOXN (X = O, S) calculated at the CCSD­(T)/aug-cc-pVTZ level were shown in Figure 1 and in excellent agreement with previous results.^12,16^ Unlike HOXH (X = O, S) which prefers a skew structure, both *trans*-HOON and *trans*-HOSN possess planar geometries. The length of the O–O bond in *trans*-HOON is 1.909 Å, significantly longer than the length of the O–O bond in H_2_O_2_, which measures 1.461 Å at the same computational level. For *trans*-HOSN, the O–S bond length (1.699 Å) closely matches that of *trans*-HOSH (1.684 Å).

**Figure 1:**
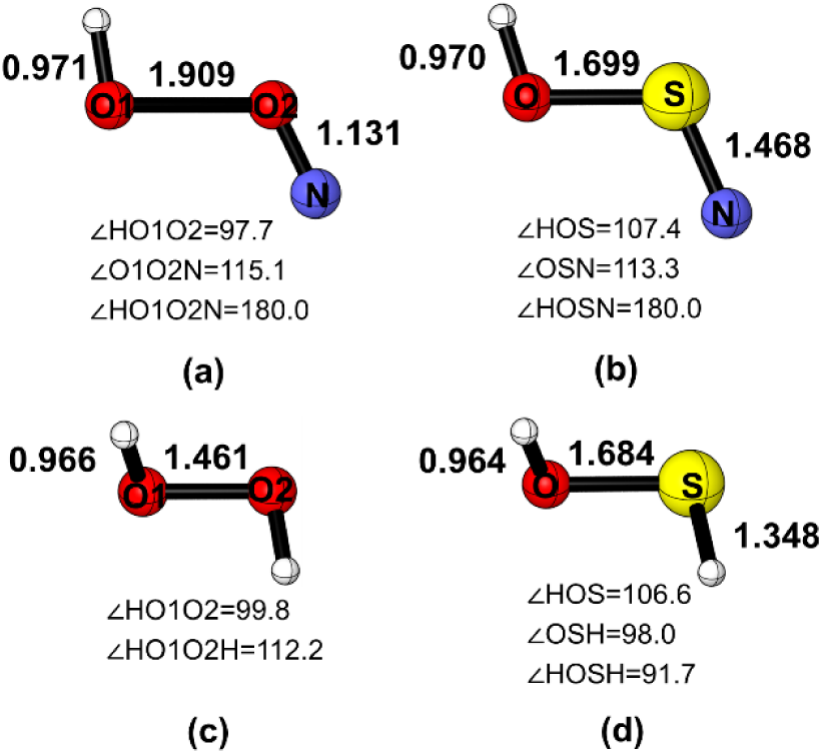
Optimized structures of (a) *trans*-HOON, (b) *trans*-HOSN, (c) H_2_O_2_, and (d) HOSH at the CCSD­(T)/aug-cc-pVTZ level.

The AdNDP method^26,27^ can describe the chemical bonding by combining the compactness and intuitive simplicity of Lewis theory with the flexibility and generality of canonical MO theory. The algorithm is a generalization of the natural bonding orbital (NBO) analysis.^27^ The chemical bonding entities in this method are *n*-center 2-electron (*n*c-2e) bonds, where *n* ranges from one (lone pair) to the maximum number of atoms in the system (completely delocalized bonding). Figure 2 presents the AdNDP orbitals and the electron occupations of *trans*-HOXN (X = O, S). In both cases, there are totally four lone pairs located on the O, X (X = O, S) and N atoms, alongside three 2c–2e bonds, including one O–H bond, one O–X σ bond, and one X–N π bond. Notably, HOON contains two 3c–2e σ bonds, while the corresponding bonds in HOSN are 2c–2e bonds. In HOON, the first 3c–2e σ-conjugated bond with two O atoms being the major contributors is predominantly the σ_O–O_ orbital, and the second one signifies the hyperconjugative interaction from lone pair electrons of the N atom to the σ*_O–O_ orbital, which is responsible for the weakening and elongation of the O–O bond. Therefore, the active space should consist of four electrons and three associated hybrid atomic orbitals in our classical VB calculations. The VB structures in the (4e, 3o) active space are presented in Figure 3.

**Figure 2:**
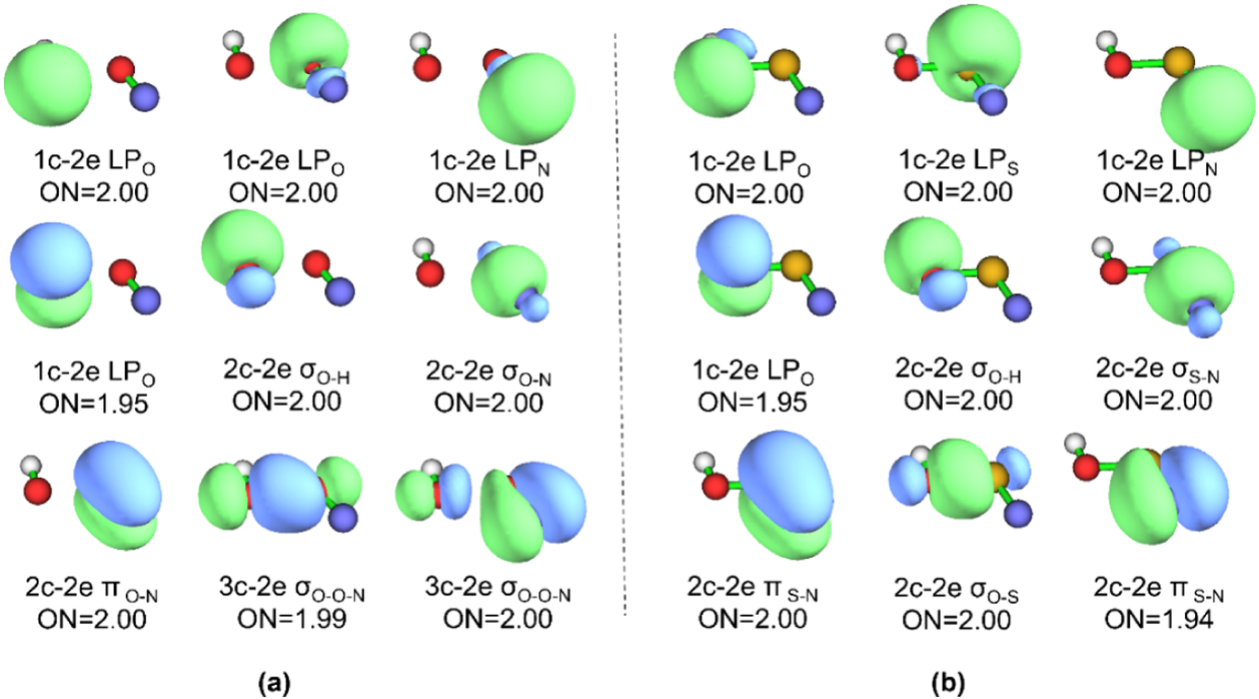
AdNDP bonding patterns for (a) *trans*-HOON and (b) *trans*-HOSN.

**Figure 3:**
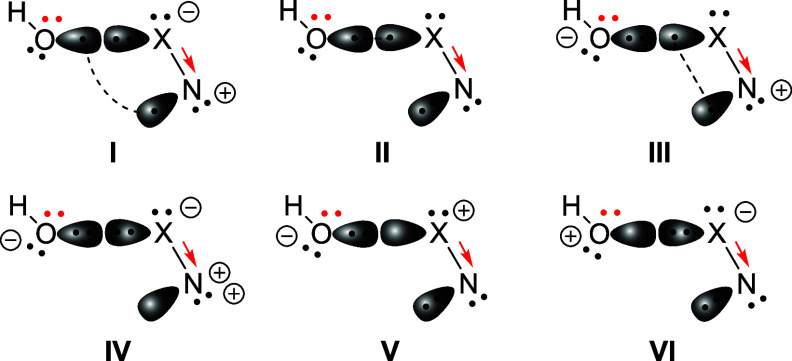
VB structure set for *trans*-HOXN (X = O, S) in the (4e, 3o) active space. Black dots correspond to σ electrons, with π electrons indicated by red dots and arrows.

### Bonding Nature of *trans*-HOON: Three-Center Four-Electron Charge-Shift Bond

Based on the six resonance structures, we performed ab initio VB computations with the BOVB approach. In Figure S1, we showed the BOVB orbitals of structure **I** for *trans*-HOON and structure **II** for *trans*-HOSN as examples. Figure 4a shows the energy curves along the O–O distance for the full ground-state wave function at the BOVB level. A notable feature of the BOVB approach is its excellent prediction of the bond dissociation energy, calculated at 7.5 kcal/mol, which is only 0.9 kcal/mol lower than the value at the CASPT2 level in (18e, 13o) active space.^12^ This indicates that the compact VB functions, involving just six configurations within the (4e, 3o) space, are sufficient to describe the HOON system. In contrast, MO-based methods usually require many more configurations in computations.

**Figure 4:**
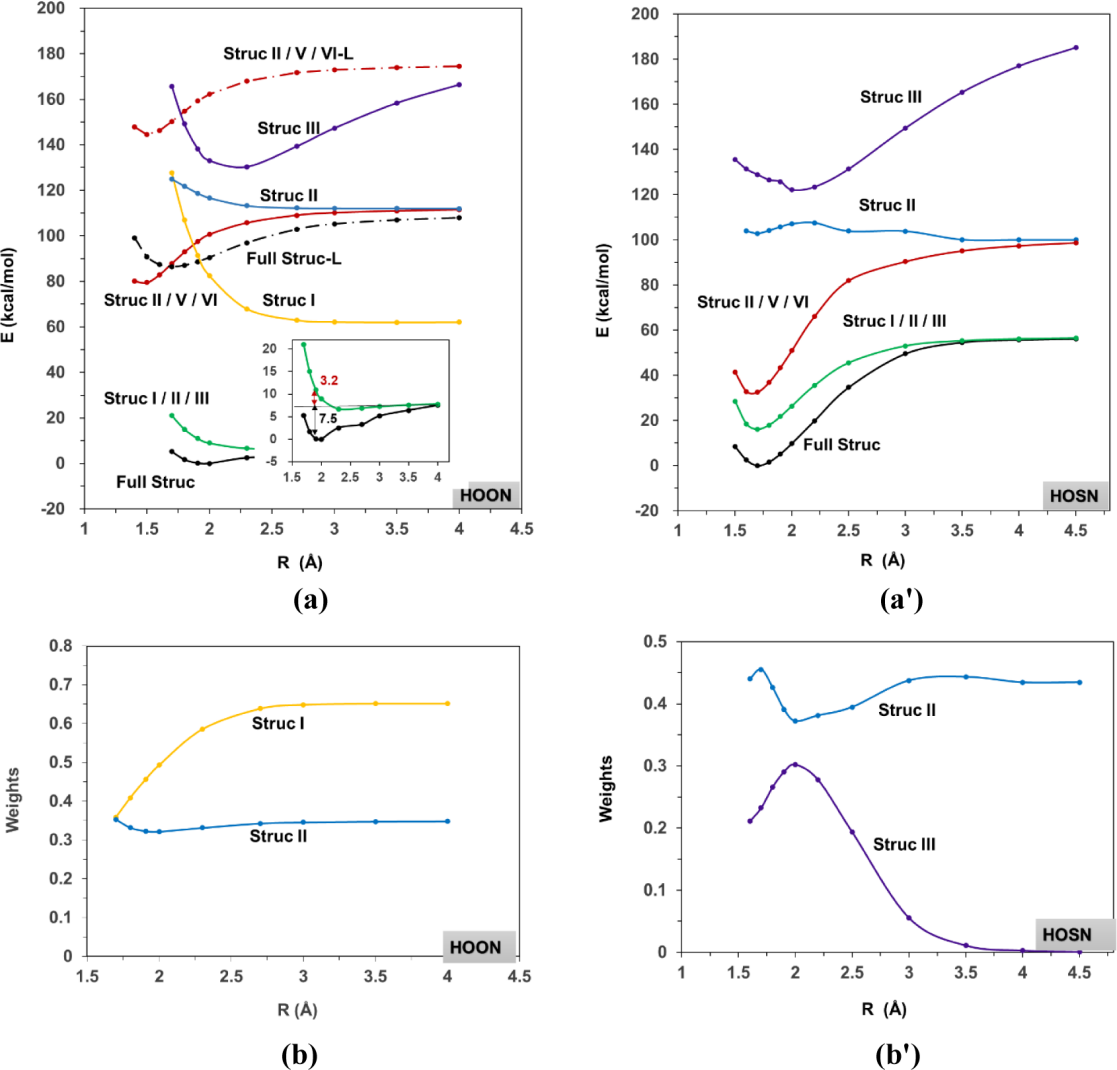
Potential energy curves and structural weight evolutions for *trans*-HOON (a, b) and *trans*-HOSN (a’, b’) along the O–O distance. Structure **I** is not shown in (a’) because its energy is much higher than those of **II** and **III**. Labels “Full Struc-L” and “Struc **II**/**V**/**VI**-L” (dashed lines in (a)) correspond to BOVB calculations where all oxygen lone pairs were rigorously localized and no dative π bond between O2 and N.

The evolution of structural weights for significant structures (those with values greater than 0.2 at the equilibrium distance) is illustrated in Figure 4b. Obviously, at the equilibrium distance, the principal structures are **I** and **II**. This is in agreement with the conclusion by Talipov *et al.* that the NO moiety in HOON most closely resembles nitric oxide (structure **I**), with some nitrene character (structure **II**).^12^ Based on their GVB calculations, Takeshita and Dunning^16^ attributed the long O–O bond in *trans*-HOON to an unusual, weakly attractive through-pair interaction (Figure 1 and 2 of ref 16) that couples the singly occupied π orbital of OH with the singly occupied π orbital located largely on the nitrogen atom. Despite the differences of interpretations between the GVB and our BOVB results being viewed through two different lenses, both studies highlight the importance of structure **I**.

Both structures **I** and **II** were also found to have the lowest energies at large interfragment distances, as the HO + NO homolytic dissociation is thermodynamically preferred. Structure **II** is not even bonded; that is, the bond dissociation energy is negative. A similar unbound covalent structure has been discussed previously in archetypal charge-shift bonds^32–36^ (e.g., F–F, HO–OH, and H_2_N–NH_2_), where the covalent-ionic resonance (or coupling) plays a dominant role. The bond weakening observed in such cases can be attributed to the inherent properties of the atoms or fragments, as described by Sanderson.^37^ In structure **II**, the O1 atom possesses one lone pair orbital and one singly occupied bonding orbital, which can overlap with the singly occupied bonding orbital of O2. While the singly occupied orbitals overlap (overlap integral S = 0.012) to form the σ O–O covalent bond, there exists three-electron Pauli repulsion (*S* = 0.075) between the lone pair orbital of O1 and the singly occupied bond orbital of O2. Similar three-electron repulsion has been discussed previously by Hiberty and Shaik,^33,38^ and we repeated it in brevity, as shown in Figure S2. Since the overlap capability of the singly occupied orbital of O1 is smaller than that of the lone pair orbital, the bonding cannot sufficiently shield the repulsion. For structure **I**, in addition to lone pair repulsion analogous to that observed in structure **II**, the three electrons in the two active orbitals on the O atoms occupy a common space and maintain Pauli repulsion. Despite more pronounced repulsive effects, structure **I** exhibits greater stability than structure **II** at equilibrium geometry. This is because it is the only neutral resonance structure, and there is the polarization preference in the NO fragment, as illustrated in principal structure **I**.

We reexamined the six VB structures and found that structures **I** to **III** all feature spin-paired covalent bonds, while structures **IV** to **VI** resemble ionic structures and possess one unoccupied orbital. When only the spin-paired covalent structures (**I** to **III**) within the (4e, 3o) active space are considered at the BOVB level, the dissociation energy remains negative (−3.2 kcal/mol). This indicates that the bonding energy in the HOON molecule is primarily derived from the resonance between these three covalent structures (**I** to **III**) and the three ionic structures (**IV** to **VI**). As a distinct class alongside the traditional covalent and ionic bonds, the charge-shift bond is characterized by significant resonance energy resulting from the mixing of the Heitler–London (HL) structure with ionic structures.^32–36^ Based on this criterion, the chemical bonding in HOON can be described as a three-center four-electron (3c–4e) charge-shift bond, which had been used to explain the stability of XeF_2_ by Braïda and Hiberty.^34^

### Origin of Charge-Shift Bond in *trans*-HOON: π Orbital-Delocalization-Enhanced Hyperconjugation

The charge-shift bond is an outcome of the mechanism necessary to establish equilibrium and optimum bonding during bond formation. To provide an electronic structure-based explanation for the above findings and the stretched O–O bond length in *trans*-HOON, we focused on the hyperconjugative interaction between the lone pair of the N atom and the σ*_O–O_ orbital. The two active electrons constituting the O–O bond are described by covalent structure **II** and ionic structures **V** and **VI**. In the language of resonance theory, the hyperconjugative interaction is represented as a correction due to the contributions of structures **I**, **III**, and **IV**. Given the significant roles of **I** and **III**, the hyperconjugation effect cannot be ignored. To further investigate this, we performed BOVB calculations using only structures **II**, **V**, and **VI** (red line in Figure 4a) to examine the situation without hyperconjugation. Interestingly, we observed an energy minimum around 1.5 Å, which is close to the O–O bond length found in HOOH. The hyperconjugation energy reaches 71.2 kcal/mol at an O–O bond length of 1.5 Å. We note that the most recent XMVB 4.0 enables geometry optimization at the VBSCF level. By utilizing structures **II**, **V**, and **VI**, we also found that the *trans*-HOON adopts a planar configuration with an O–O bond length of 1.529 Å at the VBSCF level.

In HOON, each oxygen atom has a lone pair of σ symmetry and another lone pair of π symmetry, but the *p*_π_ orbital of the nitrogen atom is vacant. The π bond between the O2 and N atoms thus arises from the dative interaction between the lone pair of π symmetry on O2 and the vacant *p*_π_ orbital of N, as shown in structure **II**. This electron donation induces a negative charge on N, which would significantly stimulate the interaction between the lone pair of σ symmetry in the planar N atom and the σ*_O–O_ orbital. To investigate the synergistic effects between σ and π components, we performed BOVB calculations with localized π electrons (i.e., no dative π bond between O2 and N by keeping the *p*_π_ orbital of N strictly vacant). The energy minimum persists around 1.5 Å with structures **II**, **V**, and **VI** (red dashed line in Figure 4a), whereas the minimum shifts to approximately 1.7 Å with all six structures (black dashed line in Figure 4a). By comparing the earlier result (1.9 Å) using delocalized π orbitals, we concluded that the π-orbital-delocalization-enhanced hyperconjugation is the governing factor for the stretched and weakened O–O bond in *trans*-HOON.

Based on the natural resonance theory,^39–41^ we plotted the lone pair of the N atom and the antibonding orbital σ*_O–O_ at 1.5 Å as shown in Figure 5a. A direct and quantitative measure of hyperconjugation can be obtained from the second-order perturbation analysis within the NBO theory,^39–41^ which generated a value of 155.4 kcal/mol. Although the NBO method tends to overestimate the hyperconjugation, it is noteworthy that both the VB and NBO methods highlight the significance of the hyperconjugative interaction in *trans*-HOON.

**Figure 5:**
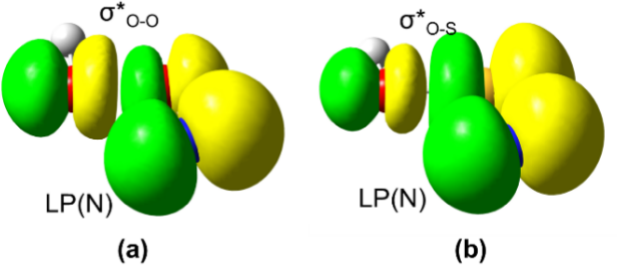
Lone pair of N atom and antibonding σ*_O–O/O‑S_ orbital in (a) *trans*-HOON and (b) *trans*-HOSN.

### Bonding Nature of *trans*-HOSN: Two-Center Two-Electron Charge-Shift Bond

For *trans*-HOSN, the bond dissociation energy calculated at the BOVB level (56.2 kcal/mol) was a little lower than that obtained at the CCSD­(T) level (62.2 kcal/mol). This discrepancy can likely be attributed to electron correlation. For example, at least the electron correlation among the π electrons in our BOVB computations was not considered.

As illustrated in Figure 4a’,b’, covalent structures **II** and **III** are dominant at the equilibrium bond distance. Similar to the case of *trans*-HOON, structure **II** is also unbound in *trans*-HOSN. Although there is three-electron Pauli repulsion between lone pair orbitals of O and the singly occupied bond orbital of S, the electrostatic interaction between OH^–^ and NS^+^ leads to an energy minimum for structure **III** at about 2.0 Å. The relative structural weight of **II** changes mildly, while the weight of **III** changes abruptly with the stretch of O–S bond. Compared with *trans*-HOON, the significance of structure **I** decreases while that of structure **II** increases in *trans*-HOSN. This may be due to the lower electronegativity of S atom than that of O, which allows the OH group in *trans*-HOSN to gain electrons.

The two active electrons constituting the O–S bond are described by covalent structure **II** and ionic structures **V** and **VI**. Given that the sum of weights of structures **II**, **V**, and **VI** amounts to 0.72 at the equilibrium bond distance, we reevaluated the bond dissociation energy in this (2e, 2o) active space, yielding a value of 66.2 kcal/mol (red line in Figure 4a’). Furthermore, the energy minimum at this level appears around 1.7 Å, which agrees well with the value (56.2 kcal/mol) in (4e, 3o) active space. Therefore, although there is considerable hyperconjugative interaction (see Figure 5b, −32.6 and −78.5 kcal/mol based on BOVB and NBO methods, respectively), the (2e, 2o) active space suffices for achieving the correct geometry for *trans*-HOSN. Based on GVB calculations, the O–S bond in *trans*-HOSN is formed by the coupling between singly occupied orbitals centered on the OH radical and sulfur atom.^16^ Therefore, our results align with those reported by Takeshita and Dunning.^16^ The O–S bond can consequently be classified as a two-center, two-electron charge-shift bond.

One may wonder why the hyperconjugation in *trans*-HOSN is much weaker than in *trans-*HOON. Both NBO and VB calculations may provide clues. On one hand, NBO calculations showed that the energy gap between the lone pair of N and antibonding σ*_O–X_ orbitals increases from 182.0 kcal/mol in *trans*-HOON to 232.2 kcal/mol in *trans*-HOSN. On the other hand, VB calculations showed that the overlaps between the lone pair of N and the atomic bonding orbitals composing the antibonding σ*_O–X_ orbital in structure **II** reduce from 0.026 and 0.205, respectively, in *trans*-HOON to 0.018 and 0.160 in *trans*-HOSN. The enlarged energy gap and the diminished overlaps ultimately result in the much reduced hyperconjugation in *trans*-HOSN than in *trans*-HOON.

### Origin of Planar Geometry

In contrast to H_2_O_2_, *trans*-HOON employs a planar configuration, even under VBSCF calculations using fully localized valence bond orbitals with structures **II**, **V**, and **VI** only. This indicates that conjugation and hyperconjugation effects are not the underlying causes. Previously, some of us elucidated the conformations of ethane, hydrogen peroxide, and hydrazine.^42,43^ Here, we employed a similar strategy (see Figure 6) to rationalize the planar configuration of HOON. Since we were unable to find an optimal skew configuration for HOON, we constrained the dihedral angles ∠HOON to match those in H_2_O_2_. At the CCSD­(T) level, the planar configuration is energetically more stable than the skew configuration by 1.6 kcal/mol. Parallel BOVB calculations employing strictly localized orbitals in structures **II**, **V**, and **VI** show a comparable result (1.2 kcal/mol). Consistent with the aforementioned geometry optimization results, neither conjugation nor hyperconjugation serves as the primary factor responsible for the planar geometry. We further decomposed the rotational process into two sequential steps. In the first step, all structural parameters except the dihedral angle ∠HOON were constrained while rotating the hydroxyl group. The resulting energy decrease of 2.4 kcal/mol is attributed to the release of steric repulsion and bond orbital rehybridization. Subsequently, full geometry relaxation (excluding ∠HOON) to the optimal rotated conformation required an energy input of 3.6 kcal/mol. Obviously, it is geometric relaxation that contributes to the planar configuration.

**Figure 6:**
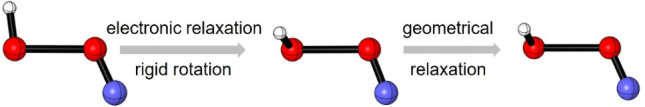
A stepwise decomposition scheme to explore the rotation of HOON.

Building on the above strategy, we continued to elucidate the origin of the planar configuration of *trans*-HOSN. The planar configuration exhibits a stabilization energy of 3.3 kcal/mol relative to the skew conformation at the CCSD­(T) level. At the BOVB level, rigid rotation results in an energy reduction of 5.6 kcal/mol, while subsequent conformational relaxation leads to an energy increase of 1.0 kcal/mol. Therefore, the skew configuration is energetically more stable than the planar configuration by 4.6 kcal/mol. As the conjugation and hyperconjugation effects have been deactivated in these BOVB calculations, the above results highlight the critical role of conjugation and hyperconjugation effects in stabilizing the planar geometry of *trans*-HOSN.

## Conclusions

The *trans*-HOXN (X = O, S) molecules have been studied using the classical ab initio VB method to gain detailed insight into the bonding nature and the underlying reasons for the long O–O bond in *trans*-HOON. Based on the results from AdNDP, the (4e, 3o) active space that incorporates the lone pair of the nitrogen atom and the σ_O–O_ orbital is essential for our VB calculations. The bonding in *trans*-HOON is primarily characterized by two covalent VB structures (**I** and **II** in Figure 3). As demonstrated by Talipov *et al.*, the NO moiety in *trans*-HOON closely resembles nitric oxide, with some nitrene character. However, neither of these structures is stable at equilibrium, as they exist at energies significantly higher than the dissociation limit. Instead, the mixing of covalent and ionic VB structures within the (4e, 3o) active space generates a considerable resonance energy, which exceeds the bonding energy itself. Consequently, the O–O bond in *trans*-HOON should be classified as a three-center four-electron charge-shift bond. The π-orbital-delocalization-enhanced hyperconjugation arising from the lone pair of the nitrogen atom and the non-Lewis antibonding orbital (σ*_O–O_ orbital) is the key factor contributing to the charge-shift bonding and the elongated O–O bond in *trans*-HOON, while the geometric relaxation contributes to the planar configuration. In contrast, for *trans*-HOSN, although hyperconjugation is also present, a (2e, 2o) active space is sufficient to achieve the correct geometry. The O–S bond can, therefore, be classified as a two-center, two-electron charge-shift bond. And conjugation and hyperconjugation effect play an important role in stabilizing the planar geometry of *trans*-HOSN.

## Supplementary Material



## Notes

BOVB orbitals of structure **I** for *trans*-HOON and of structure **II** for *trans*-HOSN at their equilibrium geometries and scheme for the three-electron repulsion in HOON (PDF)
