# Supplementary material for: Abnormally Long O–O Bond in trans-HOON: An Exemplary Charge-Shift Bond
Source: J Phys Chem A. 2025 Jun 25;129(27):6011–7. doi: 10.1021/acs.jpca.5c02743 (PMC12257518; doi:10.1021/acs.jpca.5c02743)
Supplement: Supplementary file 1 [file jp5c02743_si_001.pdf]

Supporting Information

Abnormally Long O-O Bond in *trans*-HOON: An Exemplary Charge-Shift Bond

Huaiyu Zhang,[,a] Jia Wei,[a] Rui Ma,[a] Jinshuai Song,[b] Wei Wu,[*,c] Yirong Mo[*,d]*

[a] Institute of Computational Quantum Chemistry, and Hebei Key Laboratory of Inorganic Nano-materials, College of Chemistry and Materials Science, Hebei Normal University, Shijiazhuang, 050024, China

[b] Green Catalysis Center, and College of Chemistry, Zhengzhou University, Zhengzhou, 450001, China

[c] State Key Laboratory of Physical Chemistry of Solid Surfaces, Fujian Provincial Key Laboratory of Theoretical and Computational Chemistry, and College of Chemistry and Chemical Engineering, Xiamen University, Xiamen, 361005, China

[d] Department of Nanoscience, Joint School of Nanoscience and Nanoengineering, University of North Carolina at Greensboro, Greensboro, North Carolina, 27401, United States

1. BOVB orbitals

In BOVB computations, each structure is constructed with its own set of optimal orbitals. Here we exemplify the BOVB orbitals of structure **I** for *trans*-HOON and structure **II** for *trans*-HOSN at the equilibrium distance are shown.

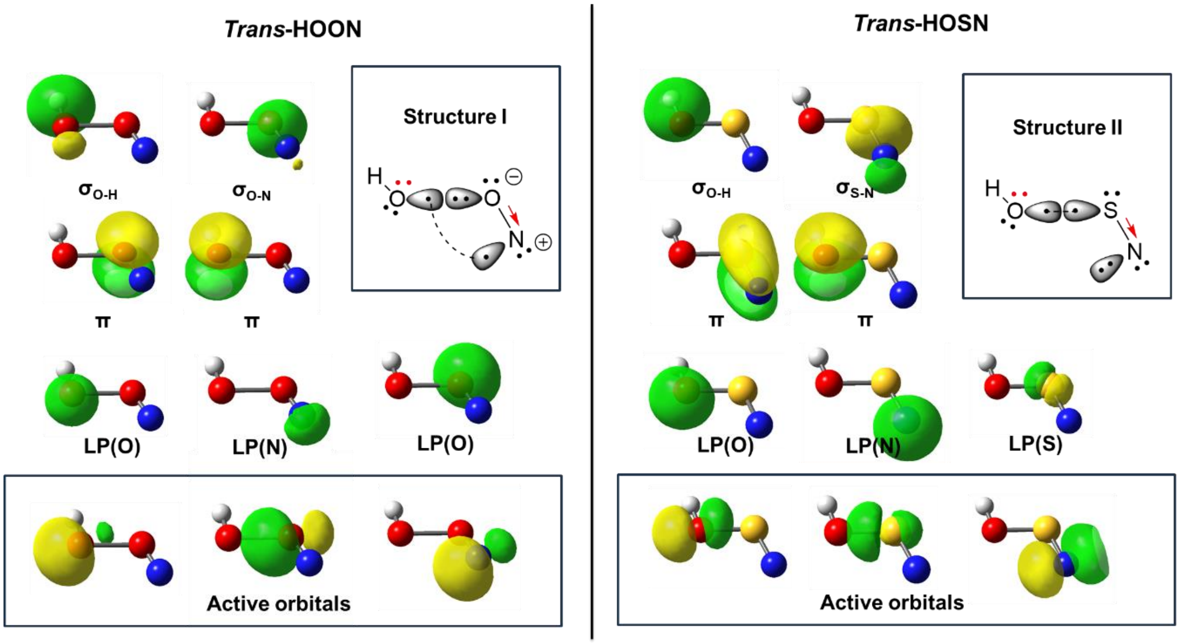

Figure S1. BOVB orbitals of structure **I** for *trans*-HOON and structure **II** for *trans*-HOSN at the equilibrium distance.

2. Three-electron repulsion in structure **II** is schematically shown in Figure S2.

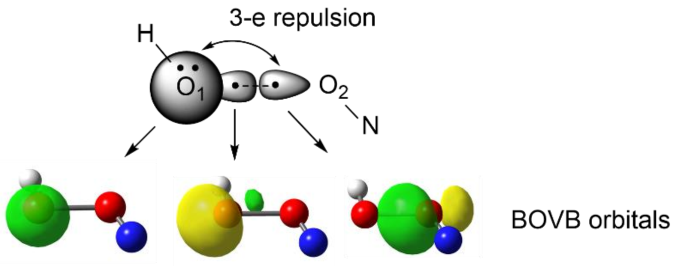

Figure S2. Three-electron repulsion in structure **II.**
